# Coconut Oil and Cardiovascular Disease Risk

**DOI:** 10.1007/s11883-023-01098-y

**Published:** 2023-03-27

**Authors:** Lukas Schwingshackl, Sabrina Schlesinger

**Affiliations:** 1 Institute for Evidence in Medicine, Medical Center - University of Freiburg, Faculty of Medicine, University of Freiburg, Breisacher Straße 86, 79110 Freiburg, Germany; 2 German Diabetes Center, Institute for Biometrics and Epidemiology, Düsseldorf, Germany; 3 German Center for Diabetes Research (DZD), Partner Düsseldorf, Munich-, Neuherberg, Germany

**Keywords:** Coconut oil, Cardiovascular, Evidence, Meta-analyses

## Abstract

**Purpose of Review:**

This narrative review summarizes the current peer-reviewed literature and mechanisms surrounding the cardiovascular health impact of coconut oil.

**Recent Findings:**

No randomized controlled trials (RCTs) and/or prospective cohort studies have investigated the effect or association of coconut oil with cardiovascular disease. Evidence from RCTs indicated that coconut oil seems to have less detrimental effects on total and LDL-cholesterol compared to butter, but not compared to *cis*-unsaturated vegetable oils, such as safflower, sunflower, or canola oil. The isocaloric replacement (by 1% of energy intake) of carbohydrates with lauric acid (the predominant fatty acid in coconut oil) increased total cholesterol by 0.029 mmol/L (95% CI: 0.014; 0.045), LDL-cholesterol by 0.017 mmol/L (0.003; 0.031), and HDL-cholesterol by 0.019 mmol/L (0.016; 0.023).

**Summary:**

The current evidence from shorter term RCTs suggests that replacement of coconut oil with *cis*-unsaturated oils lowers total and LDL-cholesterol, whereas for the association between coconut oil intake and cardiovascular disease, less evidence is available.

## Introduction

Coconut oil has been an important edible oil for the food industry for many years and is normally termed or classified as a lauric oil, a tropical oil, or a confectionery fat. The usual commercial product is either refined, bleached, and deodorized coconut oil or, more recently, virgin (unrefined) coconut oil [1].

The production of coconut oil has been increasing worldwide. The Food and Agriculture Organization (FAO) of the United Nations (UN) estimates that more than 3 million tons of coconut oil was produced in 2019, with the Philippines, Indonesia, and India being the leading producers [2]. In 2019, a global average consumption of 0.26 kg per capita/year was reported, 0.20 kg per capita/year in the USA, and 0.38 kg per capita/year in Asia (3.7 kg per capita/year in the Philippines and 2.2 kg per capita/year in Indonesia).

One of the advantages of coconut oil is its resistance to oxidation and polymerization that makes it a stable oil for cooking. Because of its high content of saturated fatty acids (SFA) (~90%) (Table 1; [3]), coconut oil has always been classified along with butter, palm oil, and animal fats as a source of SFA that should only be consumed at low levels in the diet [4, 5]. Reducing the intake of SFA is a cornerstone of dietary guidance, especially in the prevention of cardiovascular disease (CVD) [5–7], which remain the leading cause of disease burden in the world [8]. The purpose of this narrative review is therefore to summarize the latest research available on the consumption of coconut oil on risk of CVD or CVD risk markers.

**Table 1:** Composition of coconut products [3]

Coconut product	Component (per 100 g of product)						
	Water (g)	Energy (kcal)	Protein (g)	Fat (g)	Saturated fat (g)	Carbohydrates (g)	Fiber (g)
Coconut, fresh	47	354	3.33	33.5	29.7	15.2	9
Nuts, coconut meat, dried (desiccated),	3	660	6.88	64.5	57.2	23.6	16.3
Nuts, coconut cream, raw	53.9	330	3.63	34.7	30.8	6.65	2.2
Coconut oil	0	892	0	99.1	83	0	0
Shortening confectionery, coconut (hydrogenated) and/or palm kernel (hydrogenated)	0	884	0	100	91.3	0	0
Coconut milk	95	31	0.21	2.08	2.08	2.92	0

### Composition

Coconut oil is composed mainly of the SFA lauric acid (C12:0) (42 g per 100 g), but also of other SFAs, including the long-chain myristic acid (17 g per 100 g), palmitic acid (9 g per 100 g), stearic acid (3 g per 100 g), and the medium-chain fatty acids, caprylic acid (7 g per 100 g) and capric acid (5 g per 100 g), and the monounsaturated fatty acid, oleic acid (6 g per 100 g) (Table 2; [3]).

**Table 2:** Comparison of the properties of coconut oil, hydrogenated coconut oil, shortening confectionery, coconut (hydrogenated) and/or palm kernel (hydrogenated), and butter [3]

Fatty acids (g per 100 g of product)	Coconut oil	Hydrogenated coconut oil	Shortening confectionery, coconut (hydrogenated) and or palm kernel (hydrogenated)	Butter
Caprylic acid SFA C8:0	6.8	7.57	4.3	1.19
Capric acid SFA C10:0	5.39	5.54	4.3	2.53
Lauric acid SFA C12:0	41.8	44.2	35.8	2.59
Myristic acid SFA C14:0	16.7	16.4	14.4	7.44
Palmitic acid SFA C16:0	8.64	8.56	10.7	21.7
Stearic acid SFA C18:0	2.52	10.4	21.9	10
Oleic acid MUFA C18:1	6.27	0.267	2.2	20.4
Linoleic acid PUFA C18:2	1.68	0	1	1.83

*MUFA*, Monounsaturated fatty acid; *PUFA*, polyunsaturated fatty acids; *SFA*, saturated fatty acids

### Virgin vs. Refined Coconut Oil

Compared to virgin coconut oil, hydrogenated coconut oil contains a higher amount of stearic acid, and a lower amount of monounsaturated fatty acids (Table 2; [3]). Virgin coconut oil contains a higher amount of phenolic acids. The total polyphenol content of both refined and virgin coconut oil depends on environmental conditions during coconut maturation, the techniques used to extract the oil, and the extent of processing and conditions during transport and storage. Processing is usually inversely related to the amount of dietary bioactive compounds contained in the final product. The main phenolic acids found in virgin coconut oil are, in ascending order of concentration, caffeic acid, syringic acid, p-coumaric acid, vanillic acid, and ferulic acid [9, 10]. Virgin coconut oil also contains higher amounts of flavonoids, particularly flavanones, and dihydroflavonols, as well as vitamins A and E [9].

## Coconut Oil and Cardiovascular Events

### Evidence from Ecological and Observational Studies

Epidemiological evidence from indigenous populations in the Tokelau Island Migrant Study, who consume high amounts of coconut in the form of flesh or squeezed coconut cream, suggest that coconut fat was not detrimentally associated with cardiovascular health [11]. Rather, the transition of these indigenous populations to a more Western dietary pattern resulted to an increase in obesity and CVD [11, 12]. Cross-sectional studies of Kitava and Samoan populations showed similar associations as the studies of Tokelauans [13–15]. Observational studies have also been performed in several other countries such as Indonesia [16] and India [17] and findings showed no association between coconut oil consumption and prevalence of coronary heart disease risk. A recent systematic review, based on 13 observational studies, showed that consumption of coconut flesh or squeezed coconut in the context of traditional dietary patterns was not associated with adverse cardiovascular outcomes [18]. However, due to the limitations of these observational study designs (i.e., cross-sectional, case-control), which are prone to selection bias, confounding, ecological bias, and recall bias, findings need to be interpreted with caution.

### Evidence from Prospective Studies

No randomized controlled trials (RCTs) or prospective cohort studies have investigated the effect or association of coconut oil on cardiovascular events such as myocardial infarction, heart failure, or stroke. It is unlikely that an RCT will be conducted on this topic, given the high cost of hundreds of millions of dollars, the large numbers of needed participants, the long duration of the intervention with coconut oil, and, finally, the selection of the appropriate control fat. However, evidence on the association between coconut oil intake and the risk of CVD derived from prospective observational studies may be feasible.

According to the Global Burden of Diseases Nutrition and Chronic Diseases Expert Group (NutriCoDE) the mean SFA intake is approximately 10% worldwide [19] whereas in the European Prospective Investigation into Cancer and Nutrition cohort study, the mean SFA intake was ≥14% of total energy intake [20]. These data show that in many countries, SFA intake is above the recommended levels [5]. Further research is needed to help individuals to make effective changes to reduce SFA intake and to maintain these changes over a longer term. Public health nutrition policies, such as improved labelling, pricing initiatives, and improved availability of healthier foods, may yield huge advances in this research field.

## Coconut Oil and Cardiovascular Risk Factors

### Evidence from Systematic Reviews of Randomized Controlled Trials

A large network meta-analysis of 54 RCTs compared the effects of 13 different oils (safflower, sunflower, canola, hempseed, flaxseed, corn, olive, soybean, palm, coconut) and solid fats (beef fat, lard, butter) on blood lipids in generally healthy participants, and in participants at CVD risk [21^••^]. For low-density lipoprotein cholesterol (LDL-C) and triacylglycerols, no clear differences between coconut oil and unsaturated rich oils were observed (Table 3). However, a 10% isocaloric exchange of butter with coconut oil reduced LDL-C by −0.23 mmol/L (95% CI: −0.40 to −0.07), total cholesterol by −0.18 mmol/L (95% CI: −0.34 to −0.02), and improved HDL-C by 0.04 mmol/L (95% CI: 0.01 to 0.08). On the contrary, several unsaturated rich oils such as safflower, sunflower, canola, and corn oil reduced total cholesterol compared to coconut oil [21^••^]. However, in this network meta-analysis, the authors did not distinguish between the effects of virgin vs. refined coconut oil.

**Table 3:** Summary effect estimates (difference in mean per 10% isocaloric exchange) for the comparison of coconut oil compared to 12 other oils/solid fats on blood lipids based on findings by a network meta-analysis of Schwingshackl and colleagues 2018 [21^••^]

	Comparator	Total cholesterol (mmol/L)	LDL-cholesterol (mmol/L)	HDL-cholesterol (mmol/L)	Triacylglycerols (mmol/L)
**Coconut oil vs.**	Safflower oil	**0.31 (0.14, 0.48)**	0.19 (−0.01, 0.38)	**0.09 (0.05, 0.13)**	0.04 (−0.01, 0.10)
	Sunflower oil	**0.19 (0.04, 0.35)**	0.11 (−0.05, 0.27)	0.03 (0.00, 0.06)	0.03 (−0.01, 0.07)
	Canola oil	**0.25 (0.07, 0.43)**	0.13 (−0.05, 0.31)	0.03 (−0.01, 0.07)	0.03 (−0.02, 0.08)
	Hempseed oil	0.17 (−0.28, 0.62)	0.17 (−0.32, 0.65)	0.01 (−0.19, 0.22)	0.05 (−0.13, 0.22)
	Flaxseed oil	0.15 (−0.11, 0.41)	0.13 (−0.12, 0.38)	0.04 (−0.02, 0.10)	0.03 (−0.06, 0.12)
	Corn oil	**0.19 (0.03, 0.35)**	0.10 (−0.07, 0.26)	**0.05 (0.01, 0.08)**	0.04 (−0.02, 0.10)
	Olive oil	0.10 (−0.05, 0.24)	0.02 (−0.13, 0.17)	0.03 (0.00, 0.06)	0.00 (−0.03, 0.04)
	Soybean oil	0.15 (−0.01, 0.30)	0.05 (−0.10, 0.21)	**0.06 (0.03, 0.10)**	0.04 (0.00, 0.08)
	Palm oil	0.07 (−0.09, 0.22)	0.01 (−0.15, 0.16)	0.01 (−0.02, 0.04)	**0.04 (0.01, 0.08)**
	Beef fat	0.07 (−0.10, 0.25)	0.05 (−0.14, 0.24)	0.01 (−0.03, 0.05)	−0.04 (−0.09, 0.01)
	Lard	−0.11 (−0.36, 0.15)	−0.15 (−0.38, 0.09)	0.03 (−0.02, 0.07)	0.02 (−0.08, 0.12)
	Butter	**−0.18 (−0.34, −0.02)**	**−0.23 (−0.40, −0.07)**	**0.04 (0.01, 0.08)**	−0.01 (−0.05, 0.03)

*HDL*, high-density lipoprotein; *LDL*, low-density lipoprotein

Bold values: 95% CI did not overlap the null effect

Another comprehensive meta-analysis included 16 RCTs and found that higher coconut oil intake increased plasma LDL-C (0.27 mmol/L, 95% CI: 0.08 to 0.46) but also HDL-C (0.10 mmol/L, 95% CI: 0.06 to 0.15), whereas no effect was detected for triacylglycerols, body weight, body fat, and markers of glycaemia and inflammation in comparison with non-tropical vegetable oils [22^••^]. The heterogeneity between the RCTs included in this meta-analysis is high and most of the RCTs did not report on the types of coconut oil used. The information was only available for six RCTs; two RCTs used organic extra virgin coconut oil, two used refined, bleached, and deodorized oil, one used fractionated coconut oil, and one RCT used filtered coconut oil obtained by pressing dehydrated coconut pulp. Due to the limited information, the authors were unable to conduct stratified analysis by the types of coconut oil used.

### Evidence from Recent Randomized Controlled Trials

The largest RCT so far in general healthy participants on coconut oil was conducted in the UK in 2017 [23^•^]. A total of 96 participants were randomized to one of the three interventions (50g/day virgin coconut oil, extra virgin olive oil or unsalted butter) for 4 weeks. LDL-C concentrations were increased on butter compared to coconut oil (0.42 mmol/L, 95% CI: 0.19 to 0.65) with no differences in the change of LDL-C in coconut oil compared to olive oil. Coconut oil also increased HDL-C compared to butter (0.18 mmol/L, 95% CI: 0.06 to 0.30) or olive oil (0.16 mmol/L, 95% CI: 0.03 to 0.28). There were no differences in changes in weight, body mass index, central adiposity, fasting blood glucose, and systolic or diastolic blood pressure among any of the three intervention groups.

The most recent RCT was not included yet in systematic reviews because it was published recently in 2021. It included 48 participants with metabolic syndrome aged 20–50 years [24^•^]. Compared to a control group, consumption of 30g/day virgin coconut oil did not result in differences in anthropometric outcomes and blood pressure. However, virgin coconut oil improved HDL-C (0.19 mmol/L, 95% CI: 0.12 to 0.26) and triacylglycerols (−0.61 mmol/L, 95% CI: −0.91 to −0.30). On the contrary, detrimental effects were observed for LDL-C (increase by 0.57 mmol/L [95% CI: 0.35 to 0.79]), total cholesterol (increase by 0.95 mmol/L 95% CI: 0.65 to 1.26), and asymmetric dimethyl arginine (increase by 2.12 μg/L 95% CI: 0.78 to 3.45). In line with these findings, Vogel and colleagues [25] showed some improvements of extra virgin coconut oil on fasting glucose and HDL-C compared to soybean oil, but the detrimental effects on LDL-C were not confirmed. In another recent RCT, Maki and colleagues [26] showed that the consumption of 4 tablespoons per day (54 g/day) of corn oil reduced non-HDL-cholesterol compared to coconut oil. Similar to the findings described above, when virgin coconut oil (30 ml/day) was compared to an equal amount of safflower oil, an increase in total cholesterol, LDL-C, and HDL-C was observed, while no differences were detected for anthropometric outcomes [27].

Neumann and colleagues [28] investigated the impact of different meal fatty acid compositions on postprandial lipemia in metabolically healthy adults and adults at risk of CVD. Although coconut oil provoked a weaker postprandial lipemic response than cocoa butter, butter, and lard in two RCTs, another RCT did not find any differences when comparing the AUC 0–3 h of postprandial triacylglycerol concentrations after meals enriched with coconut oil, tallow, and milk fat.

The largest RCT on secondary prevention so far was conducted in India between 2009 and 2014, and included 200 patients with coronary artery disease who were randomly assigned to a coconut or sunflower oil group (15% of daily energy intake) [29]. Blood lipid profile at 3 months after 1 or 2 years did not show differences in both of the groups. Of the 200 patients, two in each intervention group underwent revascularization, which can be considered a cardiovascular outcome.

### Effects of Individual Fat Classes on Blood Lipids

Since the evidence for the effects of coconut oil on cardiovascular risk factors is limited, the impact of individual fat classes is of importance in understanding the role of coconut oil in the etiology of CVD.

A well-conducted meta-regression analysis by Mensink [30^••^] showed that when carbohydrates were isocalorically replaced (by 1% of energy intake) with lauric acid (the predominant fatty acid in coconut oil), total cholesterol slightly increased by 0.029 mmol/L (95% CI: 0.014 to 0.045), LDL-C by 0.017 mmol/L (95% CI: 0.003 to 0.031), and HDL-C by 0.019 mmol/L (95% CI: 0.016 to 0.023), while triacylglycerols decreased by −0.015 mmol/L (95% CI: −0.023 to −0.007). When carbohydrates were isocalorically replaced (by 1% of energy intake) with myristic acid, total cholesterol increased by 0.060 mmol/L (95% CI: 0.042 to 0.077), LDL-C by 0.044 mmol/L (95% CI: 0.028 to 0.060), and HDL-C by 0.021 mmol/L (95% CI: 0.017 to 0.025), and triacylglycerols reduced by −0.011 mmol/L (95% CI: −0.020 to −0.002). In addition, the isocaloric replacement of carbohydrates (1% of energy intake) with palmitic acid increased total cholesterol by 0.041 mmol/L (95% CI: 0.030 to 0.052), LDL-C by 0.036 mmol/L (95% CI: 0.026 to 0.046), and HDL-C by 0.010 mmol/L (95% CI: 0.007 to 0.013), and reduced triacylglycerols by −0.011 mmol/L (95% CI: −0.017 to −0.006). Moreover, isoenergetic substitution of palmitic acid with oleic acid lowers total cholesterol, LDL-C, and apoB concentrations [31]. In a systematic review on RCTs that focused on interventions to reduce intake of SFA, no effect was observed for systolic and diastolic blood pressure [32].

Regarding glycemic parameters, an isocaloric substitution (5% of total energy) of SFA with polyunsaturated and monounsaturated fatty acids improved biomarkers of glycemic control such as fasting glucose, HbA1c, C-peptide, and HOMA-index based on a systematic review of feeding RCTs [33]. However, there was insufficient information available to classify the subtypes of fatty acids, so these findings must be considered primarily relevant to the effects of total dietary SFA (predominantly palmitic acid).

Although no data are available for the association between coconut oil and the incidence of CVD, evidence from RCTs and prospective observational studies is available regarding the dominant fatty acid type in coconut oil, SFA. In systematic reviews of prospective observational studies, a higher total SFA intake was not associated with risk of CVD [34–37]. However, this finding needs to be interpreted with caution, since the meta-analyses were mainly based on comparisons of high versus low intakes, and are therefore less informative than findings from substitution analyses. In the pooling project including eleven American and European prospective cohort studies, for a 5% lower energy intake from SFAs and a concomitant higher energy intake from polyunsaturated fatty acids, there was an inverse association with risk of coronary events [38]. Findings from RCTs focusing on coronary events confirmed these beneficial effects of replacing SFA with polyunsaturated fatty acids [39].

## Conclusions

So far, there is insufficient evidence on the intake of coconut oil and risk of CVD to draw clear conclusions. Clinical evidence from RCTs suggested that coconut oil seems to have less detrimental effects on total and LDL-C as compared to butter, but not compared to *cis-*unsaturated vegetables oils, such as safflower, sunflower, and canola oil.

As in most countries, current SFA intake is above recommended levels; further research priorities could help individuals to make effective changes to reduce SFA intake and to maintain these changes over a longer term. Moreover, the implementation of public health nutrition policies, such as improved labelling, pricing initiatives, and improved availability of healthier foods, may yield huge advances in this research field and are of high relevance.
